# Neurobiology of Aggressive Behavior—Role of Autoantibodies Reactive With Stress-Related Peptide Hormones

**DOI:** 10.3389/fpsyt.2019.00872

**Published:** 2019-12-04

**Authors:** Henning Vaeroy, Frida Schneider, Sergueï O. Fetissov

**Affiliations:** ^1^Department of Psychiatric Research, Akershus University Hospital, Nordbyhagen, Norway; ^2^Inserm UMR1239, Laboratory of Neuronal and Neuroendocrine Differentiation and Communication, University of Rouen Normandy, Rouen, France

**Keywords:** human aggression, adrenocorticotropic hormone, oxytocin, vasopressin, autoantibodies, hypothalamic-pituitary-adrenal axis, cortisol, epitopes

## Abstract

Adrenocorticotropic hormone together with arginine vasopressin and oxytocin, the neuropeptides regulating the stress response and the hypothalamic-pituitary-adrenal axis activity, are known to modulate aggressive behavior. The functional role of the adrenocorticotropic hormone immunoglobulin G autoantibodies in peptidergic signaling and motivated behavior, including aggression, has been shown in experimental and *in vitro* models. This review summarizes some experimental data implicating autoantibodies reactive with stress-related peptides in aggressive behavior.

## Introduction

In humans, aggression has developed to become part of our defense and protection, but it may also be a symptom reflecting certain medical conditions. As a state of mind, it may be directed towards objects, animals, or human beings, without any obvious motive, and aggression could be a means of self-infliction or be a result from illegal use of drugs and anabolic steroids. It may be elicited by provocation (1, 2) and be detrimental to a person's health through stress as in e.g. cardiovascular disorders (3, 4). As stress-related, aggression involves cortisol and activity in the hypothalamic–pituitary axis (HPA axis) (5).

In his theory of the general adaptation syndrome, Hans Selye emphasized the role of the immune system following the response to stress (6). Since then, we have learned that stress is a broad category including some aversive events which can elicit an aggressive response (1), and that the immune system interferes with normal and pathological brain functioning and behavior (7). Pheromones and odors from the urine have been associated with aggressive behavior (8) and over the years, scientists have had several hypothesis such as the frustration-aggression hypothesis proposed as early as in 1941 (9). It is currently accepted that aggressive behavior can be viewed as a strategy by humans and animals to cope with stress, implying that neurobiological mechanisms involved in stress responses should underlie both physiological and pathological aggression (10–13). Studies of the HPA axis, has later linked the brain's control of cortisol secretion *via* pituitary release of the adrenocorticotropic hormone (ACTH) (14). Both deficient and increased activation of the HPA axis have been associated with aggressive behavior and Cortisol suppresses the activity of the HPA axis through a mechanism of negative feedback. Cortisol also modulates behavioral modalities including anxiety and distress (15), and diminishes the production of testosterone (16). Berkowitz (17) was convinced that high aggressive drive together with personality factors could explain aggression displacement whereas hypo-arousal-associated aggressiveness, a proposed characteristic of antisocial personality disorder, has been linked to glucocorticoid deficiency (18). In contrast, hyper-arousal-driven aggressiveness, which could be related to the acute exaggerated glucocorticoid response to stress, can be seen in conditions such as post-traumatic stress disorder (PTSD) and intermittent explosive disorder. In fact a study showed that more than twice the individuals with diagnosed intermittent explosive disorder (IED) met the PTSD criteria, compared to individuals without IED(19).

After the introduction of the neuropeptide concept (20, 21) further studies have revealed that peptide hormones are the key modulators of the homeostasis, stress response, and motivated behavior (22, 23). In this regard, not only the centrally produced, but also peripherally derived peptides can access the brain (24), including transport across the blood-brain barrier (25), and diffusion together with macromolecules via the perivascular spaces (26). The circumventricular organs in the brain, with their extensive and highly permeable capillaries, represent important sites of action of peripheral peptide hormones, e.g. the median eminence located in the vicinity of the ventromedial hypothalamic nucleus involved in the regulation of aggressive behavior (27). Thus, aggressive behavior may involve specific brain circuitries and activation of the HPA axis as a mechanism of altered response to stress, however, the biological background is so far not fully understood (28–31).

Immunoglobulins (Ig) or autoantibodies (autoAbs) reactive with neuropeptides and peptide hormones have been identified in humans and rodents showing associations of their plasma levels with aggressive or antisocial behavior, anxiety, and depression. For instance, in 2002 Fetissov et al. described IgG reactive with melanocortin peptides alpha-melanocyte-stimulating hormone (α-MSH) and ACTH in patients with eating disorders (ED) (32), results which later were followed by data showing increased plasma levels of ACTH-reactive autoAbs in subjects with increased aggressive and antisocial behavior (33). Most recently, a modulatory role of ACTH-reactive IgG in ACTH-induced cortisol secretion was demonstrated (34).

Understanding the modulatory role of autoAbs reactive with stress-related peptide hormones represents a new approach to aggressive behavior. Few studies are published on this immuno-modulated behavior, and the purpose of this review is to present the most recent knowledge integrating such autoAbs in neurobiological mechanisms of aggression.

## Subtypes of Aggressive Behavior

There are long traditions of claiming that aggression falls into proactive or reactive types and that the basis for aggressive behavior is to inflict harm (12). Human aggression varies from purely reactive cases with unplanned fighting and strong emotions, to purely proactive, premeditated, and deliberate efforts to harm (35).

Reactive aggression is a response to a threat or a frustrating event, with the goal being only to remove the provoking stimulus. Reactive aggression is always associated with anger, as well as with a sudden increase in sympathetic activation and a failure of cortical regulation. In animals, reactive aggression is typically a response by the defender without any proactive elements (35), such as when a fight concerns food, whereas proactive aggression is seen rarer in most species.

Proactive aggression may refer to a planned attack with a purpose driven by an external or internal reward, and the proactivity is characterized by attention to a consistent target, and often by a lack of emotional arousal. Psychologists often distinguish between two different types of aggression, impulsive and instrumental. Impulsive or affective aggression with strong anger is not planned and it usually occurs during the heat of the moment, whereas in instrumental or predatory aggression, the aggressive behavior is goal oriented and thus normally well planned.

In this review, focus is set on the type of aggression seen in criminals sentenced to imprisonment due to their impulsive violent and extreme antisocial actions, but also where there are elements of both proactivity and premeditation, as well as of impulsivity and other personality issues. Consequently, some forms of aggressive behavior can be difficult to classify as being either one or the other, since an analysis of the kind of aggression observed in practice, often contains elements from various defined categories.

### Hypothalamic-Pituitary-Adrenal Axis

The HPA axis refers to the interaction between the hypothalamus, the pituitary gland and the adrenal cortex, and the secretion of hormones involved in the stress response. This interaction is important for the early development and later consolidation of human behavior.

Neuronal co-localization of functionally related peptides is important for an immediate physiological response in which more than one transmitter participates. Neuropeptides, normally involved as a part of long-term response to stress, e.g. a trauma or an allergic- or inflammatory reaction, need more time to upregulate than classical neurotransmitters (36). Corticotropin-releasing hormone (CRH) links the HPA axis (14) to the brain's response with stress required behavior, and its activity may thus influence anxiety and stress reactions (15). For human beings, the impact of stress already experienced during a child's early rearing environment may influence the development of later psychopathology and possibly identify hormonal substrates related to behavioral changes as the child gets older (37). Recent data have revealed that the HPA axis and associated stress-related behavior can be influenced by immunoglobulins or natural autoAbs reactive with peptide hormones involved in regulation of the HPA axis activity (34). Furthermore, experimental studies have shown enhanced activation of the hypothalamic paraventricular nucleus and amygdala in glucocorticoid-deficient rats after exposure to the resident intruder (RI) stress protocol (33, 38).

## Corticotrophin-Releasing Hormone

Specific neurons of the paraventricular nucleus (PVN) of the hypothalamus secrete CRH in response to stress. Under physiological conditions, its secretion varies during the 24 h cycle of the day; being highest in the morning and lowest during the night. The stimulation of ACTH secretion into the blood stream leads to the release of cortisol from the adrenal cortex (39). This is an automatic regulation based on negative feedback so that the blood levels of cortisol shuts down the relevant CRH release activity in the hypothalamus, thereby preventing CRH levels from becoming too high (40). It is related to aggressive behavior as part of the stress response (41).

## Adrenocorticotrophic Hormone 

ACTH consists of 39 amino acids and is a peptide originating from the precursor pro-opiomelanocortin (POMC) (42), synthetized mainly in the pituitary and in the brain. CRH stimulate the synthesis and secretion of ACTH and act in synergy with the central nervous modulatory effects of arginine vasopressin (AVP), releasing stored ACTH from corticotropic cells. ACTH binds to the melanocortin type 2 G protein-coupled receptor (MC2R) expressed in the fasciculate and reticular zones of the adrenal cortex (39, 43), and triggers intracellular signaling pathways regulating the adrenal cortisol production. Acute administrations of ACTH fragments increase fighting in mice, independently of corticosterone secretion (44), but ACTH injections in isolated mice may also decrease their aggressiveness (45). It was shown that there is a link between ACTH and aggressive behavior (46), and recently this link has been strengthened through studies on ACTH autoAbs (34).

## Cortisol

Cortisol is a steroid and the body's main stress hormone, released from the adrenal cortex. One of the first studies described a model in which the HPA axis was linked to aggression (47) and later, cortisol and aggression were seen in wrestlers who after fighting showed an increased level in serum cortisol (48). Cortisol is known from general physiology to be released during stress (49), and it contributes positively to the hormonal balance throughout the body, and most of our cells have cortisol receptors. Examples of cortisol functions are control of blood sugar levels, regulation of metabolism, anti-inflammatory effects, help to forming our memory, and depression (50).

## Melanocyte-Stimulating Hormone

The peptide hormone and neuropeptide melanocyte-stimulating hormone (MSH), is produced by the brain and pituitary and consists of α-MSH, β-MSH, and γ-MSH, which are in the family of melanocortin peptides. The sequence of α-MSH consists of the 13 first amino acids of the ACTH molecule with antibody cross-reactivity as a consequence since antibodies to ACTH and α-MSH are not specific and will detect POMC, but only to an unknown degree (51).

Experiments on male mice showed that when a dominant/subordinate pair was injected 15 min before the testing with α-MSH, the attacks on the α-MSH-treated animal were more frequent compared to when the MSH was administered 24 h before testing (52) indicating that α-MSH increases aggressive behavior. In the context of externalizing behavior, α-MSH involvement in stress (32) and aggression has been associated with melanocortin peptides since injection of α-MSH or ACTH fractions (amino acids 4–10) (45) induced aggression in mice. In addition, the melanocortin peptide pharmacophore also seems necessary for the pro-aggressive ACTH effects.

## Oxytocin

Oxytocin (OT) and AVP are both nine amino acid peptide hormones (53) and their sequences differ by two amino acids (54). OT is acting as a neuromodulator in the brain regulating social and sexual behavior. It is involved in anxiety and stress response, and in aggression (22, 55, 56). OT has protective effects against stress and studies have shown that it modulates neural circuitry for social cognition and fear in humans (57), and may disrupt the common output from the amygdala to the rat brainstem effector sites of the autonomic nervous system (58). Intracerebral OT modulation is known to inhibit stress-induced activity of the HPA axis (59, 60), causing behavioral and neural effects such as reduced anxiety (61). Administration of OT with concomitant social support during stress exposure provides the lowest cortisol response and an anxiolytic effect (62).

## Arginine Vasopressin

AVP has several peripheral and central functions, but relevant to this review regarding aggressive behavior, AVP functions as a neuromodulator. Its role in the central nervous system (CNS) seems to depend on the region in which AVP is released, including modulation of aggressive behavior (22, 56, 63, 64). AVP and CRH are found to co-exist in CRH nerve terminals (65, 66), and together with CRH, AVP strongly potentiates its ACTH-releasing activity (67, 68). As to the regulation, the function of the HPA axis has shown that in acute stress, CRH is a major player causing increased ACTH secretion, whilst in chronic stress; AVP modulation takes over as the main stimulator of ACTH release (69).

During an RI test, release of AVP, specifically in the hypothalamic mediolateral septum, was found to regulate intermale aggression in laboratory rats specifically bred for low (LAB)- or high (HAB)- anxiety‐related behavior (63). During the test exposure, LAB residents showed more aggression than the HAB residents, and the septal AVP release was found decreased in high-aggressive LAB rats compared to HAB males. Studying the patterns of AVP release within the hypothalamic mediolateral septum in the two respective groups of rats, revealed that changes in AVP release varied with intermale aggressive behavior. Thus, high levels of aggressive behavior, as seen in LAB residents, were associated with decreased release of AVP in the septum. On the other hand, the low levels of aggression found in HAB residents were associated with an increase in septal AVP release (63). Furthermore, during exposure to a non-social stressor, LAB rats responded with a stronger rise in plasma ACTH compared with HAB rats (70–72), reflecting a generally lower stressor susceptibility in the latter group, and at the same time, presenting a low trait anxiety. These findings are somewhat in line with the evidence that innate anxiety is inversely related to the level of intermale aggressive behavior (46).

## Immunoglobulins

Ig are natural antibodies produced by B1 cells, including the processes in germ-free animals after activation by both T cell-dependent and independent mechanisms (73). Ig's are divided into five classes or isotypes of which IgG is the most common type of antibody.

An autoAb is an antibody directed against one or more of the individual's own proteins. The natural autoAbs are part of normal physiology of the innate and adaptive immune defense, and may in addition be involved in several homeostatic functions (74), e.g. in removal of old erythrocytes (75), and fighting any invaders or toxins in the body, including *e.g.* bacteria and viruses. The various IgG autoAbs are relatively stable throughout life as opposed to IgG-reactive to bacterial antigens, demonstrating individual differences which in turn increase as we get older (76). Natural autoAbs of immunoglobulin M (IgM), IgG and immunoglobulin A (IgA) classes are present in all human beings without health problems (74, 77), they are polyreactive, and bind with different affinities to a variety of unrelated antigens, including those from micro-organisms.

Although the functional role of peptide hormone-reactive autoabs still needs to be further clarified, it appears that autoabs play a role in the transportation of peptide hormones and cytokines (78–81), and they seem to protect these peptides from degradation by plasma enzymes, thereby preserving biological activity (82).

## ACTH-Reactive Autoabs

The few studies on ACTH IgG autoAbs in human aggression shown in **Table 1** are those we are left with after thorough research in available databases.

**Table 1:** Selected studies on ACTH-reactive IgG autoAbs and human aggressive behavior.

Year	Title	Authors	Conclusive comments
2018	“Autoantibodies reactive to ACTH can alter cortisol secretion in both aggressive and non-aggressive humans”.	Vaeroy, et al (34)	“ACTH-reactive plasmatic IgGs exhibit differential epitope preference in controls and violently aggressive subjects. IgGs can modulate ACTH-induced cortisol secretion” and the stress response. There were different epitopes between non-aggressive and violent criminals
2013	“Corticotrophin (ACTH)-reactive immunoglobulins in adolescents in relation to antisocial behavior and stress-induced cortisol response”.	Schaefer, et al. (83)	“High total and free ACTH IgG are associated with higher antisocial behavior scores in boys. In girls, antisocial behavior is associated with low free ACTH IgG levels. Stress-induced cortisol release is associated with free ACTH IgG in boys and with total ACTH IgG in girls. ACTH IgG levels are related to antisocial behavior and HPA axis response to stress in adolescents”.
2006	“Aggressive behavior linked to corticotrophin-reactive autoantibodies”.	Fetissov, et al. (33).	“High levels of ACTH-reactive autoAbs and altered levels of oxytocin- and vasopressin-reactive autoAbs in aggressors may interfere with the neuroendocrine mechanisms of stress and motivated behavior. A new biological mechanism of human aggressive behavior” (33) is suggested.

It has been shown that autoAbs reactive with peptide hormones of the HPA axis are naturally present in rodents and in healthy humans (84), and may contribute to a more general regulation of motivated behavior, emotion, and stress-response.

Results from RI tests have shown an ACTH increase only for the intruders, but not in the aggressive residents (85). Reduced and increased stimulation of the HPA axis are to be linked to aggressive behavior (85), possibly reflecting a coping strategy to stress exposure (11, 12). With this in mind, increased plasma levels of IgG ACTH autoAbs have been found in perpetrators with increased aggressive and antisocial behavior, thereby suggesting a possible association (33). Experiments on violent criminals and non–violent individuals describe elevated levels of ACTH autoAbs which may block ACTH secretion and an instant cortisol release during stress. The blocking and non-blocking IgG effects (34) have confirmed a role for ACTH autoAbs in the HPA axis response, but at the same time makes it less likely that aberrant IgG modulation of ACTH induced cortisol response can be causal for violent aggression.

A link between autoAbs and behavior (33) is supported by a study on the general population which found increased levels of ACTH-reactive IgG in adolescent males with antisocial behavior. Psychological stress-induced cortisol release in adolescents has been found to be negatively associated with anti-ACTH IgG levels (83) and hence, it is possible that some ACTH-reactive IgG may have the analogue blocking properties as described, thereby preventing ACTH-induced activation of cortisol.

**Figure 1:**
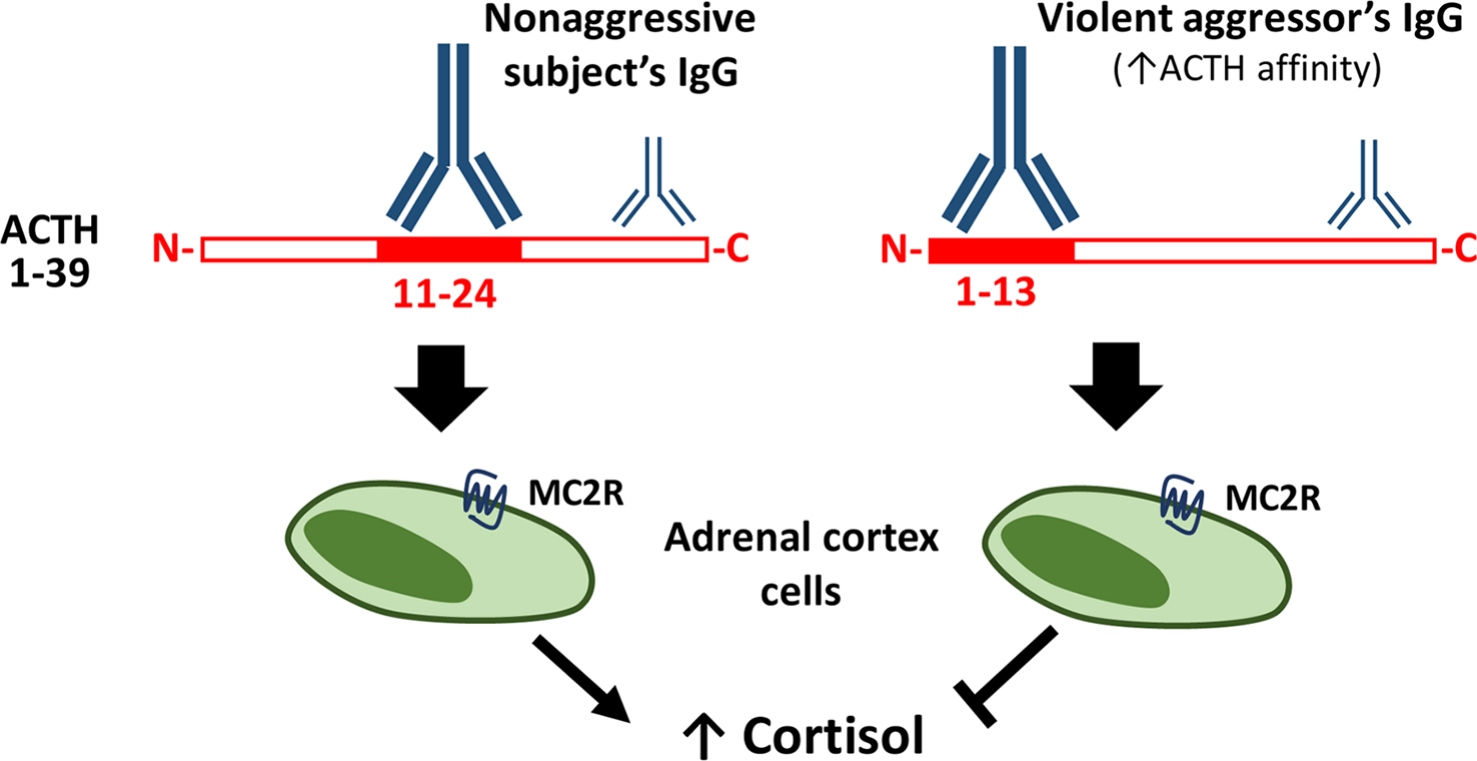
Plasma ACTH-reactive IgG modulate ACTH-induced cortisol secretion. Legend to **Figure 1**. ACTH-reactive IgG are naturally present in human plasma and modulate ACTH-induced cortisol secretion depending on the ACTH binding epitope. In the non-aggressive subjects IgG bind mainly the central part (11–24) of ACTH, containing the MC2R pharmacophore, while IgG in violent aggressors display increased affinity for ACTH and bind mainly its N-terminal part (1–13). Some IgG from both the non-aggressive and aggressive study persons have both been shown to prevent ACTH-induced cortisol secretion from the adrenal cortex cells, such inhibitory effect was associated with low IgG binding to the central ACTH part (11–24), i.e. similar to the binding pattern characterizing IgG of violent aggressors (34).

In the latest study on aggression and ACTH autoAbs, ACTH-reactive IgG were found elevated in prisoners sentenced for common crimes, but not in the extremely violent aggressors (34). An ACTH epitope overview showed that IgG binding for the cortisol responders in the group of non-aggressive controls, occur at the ACTH sequence containing the MC2R pharmacophore KKRRP (amino acids 11–24). Violent aggressors showed no binding to the ACTH amino acids 11–24, but instead to the amino acids 1–13, a section containing the melanocortin pharmacophore HFRW (34) (see **Figure 1**).

Some previously mentioned associations between ACTH autoAbs and α–MSH (32) and the blocking and non-blocking properties of the ACTH autoAbs in behavioral conditions (33) have been confirmed in the latest study, and a further step has been made, linking different epitope binding sites to non-aggressive subjects and extreme violent aggressors (34).

A lower association rate of IgG for ACTH, found in boys with antisocial behavior indicate that such autoAbs are different, not only by their plasma levels, but also possibly by their molecular structure (83). Consequently, since questions regarding potential differences in molecular structure still have to be clarified, final conclusions are premature

In a recent study applying the RI test, ACTH IgG injections from severely aggressive criminals shortened the latency for the first attack without affecting the total number of the resident's attacks (34). In addition, aggressive behavior was reduced in resident mice receiving ACTH together with IgG from non-aggressive controls (34). Other studies on rodents indicate that a rapid positive feedback exists, in which a social challenge or stressors unrelated to aggression can activate the HPA axis (86), (87).

It has been found that administration of ACTH alone, peripherally in resident mice, did not alter their aggressive behavioral expression significantly, but when co-injecting ACTH together with IgG from violent aggressors, the latency of the first attack was reduced without affecting the total number of attacks (34). Furthermore, the aggressive behavior was reduced in resident mice who received the combination of ACTH and IgG from non-aggressive controls. Such behavioral responses are supportive for a role where peripheral IgG regulates both impulsive and defensive aggressive behavior (34), and applying this model on healthy individuals, a result could be that plasma IgG may suppress natural aggressiveness (34).

## OT-Reactive autoAbs

Relevance of autoAbs reactive with OT to human behavior has been shown by statistically significant correlation between levels of anti-OT IgG in plasma and conditions with symptoms of anxiety (88). Plasma anti-OT IgM has been found to correlate with interceptive awareness, maturity fears (89). Lower plasma levels of OT reactive IgG autoAbs were also found in patients suffering from moderate levels of major depressive disorder, levels which correlated negatively with the Montgomery and Aasberg Depression Rating Scale (MADRS) (90).

Elevated levels of IgM autoAbs against OT are found in male subjects with conduct disorder (CD) and in prison inmates as compared with non-aggressive healthy controls (33). Considering OT's modulatory role in the HPA axis, increased levels of OT-reactive autoAbs may possibly interfere with OT-mediated inhibition of HPA axis during stress response in the hypo-arousal type of aggressive behavior frequently characterizing CD. Various levels of OT and AVP reactive autoAbs in patients with CD suggest that the observed changes may result in an increased AVP to OT ratio and consequently to aggressive and antisocial behavior. Furthermore, significant changes in the respective levels of OT- and AVP-reactive autoAbs may represent a factor influencing the central mechanisms behind aggressive behavior (33).

## AVP-Reactive autoAbs

Studies of autoAbs in mild and moderate depression have shown that mood changes can be associated with changes in antibody levels. However, such changes were not seen in binding the affinity of OT- and AVP-reactive autoAbs. Moreover, the levels of AVP-reactive autoAbs are associated with cortisol secretion (90). Low levels of total plasma AVP-reactive IgG autoAbs have been found in a greater proportion of depressed patients, and the free AVP IgG autoAbs showed positive correlation with plasma cortisol after physical activity (90). Similarly to anti-OT IgG, plasma levels of AVP-reactive IgG correlated with symptoms of anxiety and somatization (88) and that the levels of CRH-reactive IgG in plasma of healthy subjects correlate positively with obsessive or hypochondriac behavior, but negatively (as anti-AVP IgG) with somatization (88).

The central effects, including behavior, of AVP is as a neuromodulator. In male subjects with CD, both plasma levels of IgG and IgM classes of autoAbs reactive with AVP, have been found lower than in non-aggressive controls, but in some prison inmates, anti-AVP IgG levels were found elevated (33). If a role of the autoAbs is to protect and transport the peptide, such changes of levels of AVP-reactive autoAbs, would signify a diminished AVP-modulatory activation of the HPA axis and thus, being in agreement with the hypo-arousal theory of antisocial and aggressive behavior (91) in CD (33).

## α-MSH-Reactive autoAbs

Recent data suggest that α-MSH autoAbs can interfere with normal signal transduction in the melanocortin type 4 receptors, involved in the regulation of feeding behavior (32, 92). AutoAbs reacting with α-MSH could play a pathogenic role in psychiatric-behavioral problems in subjects with ED (93), but the presence of α-MSH- and/or ACTH- reactive autoAbs does not imply the presence of dysfunctional feeding behavior, the molecular properties of such autoAbs underlie their physiological or pathogenic role (93). Stress and gut microbiota composition may represent common denominators for production of α-MSH- and ACTH- reactive antibodies with various binding properties (80, 93, 94).

Whether or not autoAbs can pass across the blood–brain barrier (BBB) or reach the brain possibly *via* circumventricular organs, is a matter of controversy. It has been claimed that certain regulatory peptides may cross the BBB in both directions (95) and that such passing can be blocked by corresponding antibodies (96). There is a study (97) showing that the tritiated synthetic ACTH (4–10) analogue, Met-Glu-His-Phe-Pro-Gly-Pro has been detected in brain extracts at two time points (97), which according to the hypothesis suggests penetration into the brain tissues (97). Thus, further evidence is needed to strengthen any claims that the BBB function allows crossing of autoAbs, including supportive evidence of their potential modulatory role in peptide transport to the brain.

## Conclusions

Since the initial finding linking aggressive behavior to corticotrophin autoAbs by showing their high plasma levels in prisoners and in subjects with CD, there has been a development. Indeed, ACTH IgG were significantly associated with antisocial behavior and the HPA axis response in stressed adolescents. As such, higher plasma levels of ACTH IgG were present in boys with increased antisocial behavior. Girls however, showed a different picture, where the antisocial behavior was associated with low ACTH IgG levels. Moreover, recent studies were able, at least partly, to clarify the molecular mechanisms underlying an observed link between ACTH autoAbs, stress response, and aggressive behavior. It was found that in some subjects that ACTH IgG may prevent ACTH-induced cortisol secretion i.e. may disrupt a normal HPA activation. Such IgG blocking properties were associated with specific ACTH binding epitopes which were more prevalent in prisoners who had committed acts of violent aggression. Furthermore, experiments in mice showed ability of plasmatic IgG of aggressive subjects to facilitate ACTH-induced attacks in the resident-intruder test. Taken together, these data support a mechanistic role of ACTH-reactive autoAbs in the stress-related aggressive behavior. Moreover, since autoAbs reactive to others than ACTH stress-related peptides, such as OT and VP, were found at different levels in subjects with various neuropsychiatric disorders, it is likely that they may also modulate stress-induced aggressive behavior. Further studies should clarify the functional effects and origin of such autoAbs aiming at a better understanding of the neurobiological mechanism of aggressive behavior.

## Limitations and Strengths

A limitation of this review is the low number of studies published on immunobiology of human aggression including peptide hormone-reactive autoAbs. Supportive for further research on ACTH IgG in aggression is that results from studies in other clinical conditions and disorders have underlined the functional significance for autoAbs also in a topic like aggressive behavior. In this regard, we believe that recent data from studies on the microbial antigens and peptide hormone cross-reactivity as well as the gut microbiota-brain axis will influence future design of studies.

## Author Contributions

All authors listed have made substantial, direct, and intellectual contribution to the work and approved it for publication.

## Conflict of Interest

The authors declare that the research was conducted in the absence of any commercial or financial relationships that could be construed as a potential conflict of interest.
